# Exercise Promotes Tissue Regeneration: Mechanisms Involved and Therapeutic Scope

**DOI:** 10.1186/s40798-023-00573-9

**Published:** 2023-05-06

**Authors:** Chang Liu, Xinying Wu, Gururaja Vulugundam, Priyanka Gokulnath, Guoping Li, Junjie Xiao

**Affiliations:** 1 Institute of Geriatrics (Shanghai University), Affiliated Nantong Hospital of Shanghai University (The Sixth People’s Hospital of Nantong), School of Medicine, Shanghai University, Nantong, 226011 China; 2 Cardiac Regeneration and Ageing Lab, Institute of Cardiovascular Sciences, Shanghai Engineering Research Center of Organ Repair, School of Life Science, Shanghai University, Shanghai, 200444 China; 3 Biologics Development, Sanofi, Framingham, MA USA; 4 Cardiovascular Division of the Massachusetts General Hospital and Harvard Medical School, Boston, MA 02114 USA

**Keywords:** Exercise, Tissue regeneration, Muscle stem cells, Neural stem cells, Cardiomyocytes, Molecular mechanism, Regenerative therapy

## Abstract

Exercise has well-recognized beneficial effects on the whole body. Previous studies suggest that exercise could promote tissue regeneration and repair in various organs. In this review, we have summarized the major effects of exercise on tissue regeneration primarily mediated by stem cells and progenitor cells in skeletal muscle, nervous system, and vascular system. The protective function of exercise-induced stem cell activation under pathological conditions and aging in different organs have also been discussed in detail. Moreover, we have described the primary molecular mechanisms involved in exercise-induced tissue regeneration, including the roles of growth factors, signaling pathways, oxidative stress, metabolic factors, and non-coding RNAs. We have also summarized therapeutic approaches that target crucial signaling pathways and molecules responsible for exercise-induced tissue regeneration, such as IGF1, PI3K, and microRNAs. Collectively, the comprehensive understanding of exercise-induced tissue regeneration will facilitate the discovery of novel drug targets and therapeutic strategies.

## Key points

1. The protective effect of exercise on the regeneration of skeletal, muscular, vascular, and nervous system depends on exercise-induced stem cell/progenitor cell activation.
2. Exercise-promoted stem cell activation could be attributed to several factors such as signaling pathways, oxidative stress, metabolic factors, and non-coding RNAs.
3. Pharmacologically targeting the key regulators of exercise-induced stem cell activation has shown therapeutic potential.

## Introduction

**Fig. 1:**
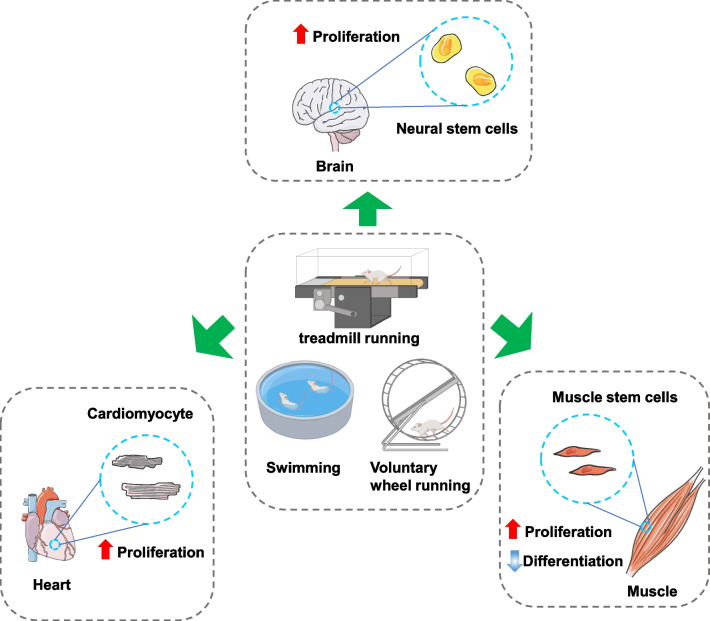
Different types of exercises and their effects on tissue regeneration

Exercise has an overall protective effect on the human body and promotes tissue regeneration, which includes cardiac regeneration [1, 2], neural regeneration [3], and muscle regeneration [4]. Studies in mice have also shown that different types of exercise, including swimming, voluntary wheel running, and treadmill running, exert specific effects on cell proliferation and tissue regeneration (Fig. 1). The most commonly used rodent exercise models include aerobic exercises such as swim training (2–4 weeks, a ramp protocol from 10- to 90-min sessions twice a day, 5–7 days/week), treadmill running (2–6 weeks, a ramp protocol from 10 min/day to 60 min/day, 5–7 days/week), and voluntary wheel running (4 days–8 weeks) [5, 6]. The variations in the types and duration of different exercises often have different stimulus effects (Table 1).

**Table 1:** Different types of exercises and their effects on regeneration

Exercise types	Organ	Affected cell types	Effects	Molecular mechanism	References
4-week treadmill training	Muscle	Muscle satellite cells	Exercise induces MSC activation and protects proliferative MSCs against exhaustion	MAPK pathway	7
5-week treadmill running	Muscle	Muscle stem cells	Endurance exercise promotes self-renewal and inhibits differentiation in satellite cells	Metabolic reprogramming and respiratory inhibition	^9^
3-week voluntary wheel running	Muscle	Muscle stem cells	Exercise accelerates muscle repair in old animals and improves old MSC function	Activation of cyclin D1 represses TGFβ signaling	^4^
4-month treadmill running	Muscle	Muscle stem cells	The numbers of CD34+ /integrin-α7+ MSC and proliferating cells in the muscles and bone marrow were enhanced by exercise	AKT pathway that is activated by an adiponectin/AdipoR1 axis	^8^
2-week treadmill training	Muscle	Fibro-adipogenic Progenitors	Exercise induces FAP senescence and muscle regeneration	AMPK	^13^
4-day voluntary wheel running	Brain	Neural precursor cells and platelets	Running induces increase in precursor cell proliferation	Platelet factor 4	^12^
7-day voluntary wheel running	Brain	Neural precursor cells	Running promotes both the proliferation and cell cycle exit of DCX(+) type-3 precursors	Notch1 activity	^35^
3-week voluntary wheel running	Brain	PVR neural stem cells	Voluntary exercise significantly increased NSC	Growth hormone	^37^
4-night voluntary wheel running	Brain	Neural precursor cells	Exercise recruits hiROS NPCs into proliferation	ROS and Nox2	^3^
Voluntary wheel running	Brain	Neural precursor cells	Exercise enhances adult hippocampal neurogenesis	BDNF	^36^
6-week treadmill running	Brain	Oligodendrocyte precursor cells	Enhanced oligodendrocyte precursor cells proliferation	N/A	^38^
8-week voluntary wheel running	Heart	Endogenous cardiomyocyte	Exercise increases birth of new cardiomyocytes	miR-222	^56^
3-week swimming	Heart	Endogenous cardiomyocyte	Exercise induces proliferation of cardiomyocytes	miR-17-3p	^60^
3-week voluntary wheel running or swimming	Heart	Endogenous cardiomyocyte	Exercise induces proliferation of cardiomyocytes	miR-222	^57^
4-week swimming	Heart	Endogenous cardiomyocyte	Exercise induces proliferation of cardiomyocytes	N/A	^61^
2-week swimming	Heart	Endogenous cardiomyocyte	Exercise induces proliferation of cardiomyocytes	C/EBPβ and CITED4	^62^
3-week swimming	Heart	Endogenous cardiomyocyte	Exercise induces proliferation of cardiomyocytes	LncCPhar, C/EBPβ, and ATF7	^70^
4-week swimming	Heart	Endogenous cardiomyocyte	Exercise induces proliferation of cardiomyocytes	C/EBPβ, ADAR2, and miR-34a	^58^

ADAR2: adenosine deaminase acting on RNA 2; AdipoR1: adiponectin receptor 1; AMPK: AMP-activated protein kinase; ATF7: activating transcription factor 7; BDNF: brain-derived neurotrophic factor; CITED4: CBP/p300-interacting transactivators with E [glutamic acid]/D [aspartic acid]-rich carboxyl-terminal domain 4; DCX: doublecortin; FAP: fibro-adipogenic progenitors; mTOR: mammalian target of rapamycin; Notch 1: notch receptor 1; NPC: neural precursor cell; NSC: neural stem cell; Nox: NADPH-oxidizing enzyme; PVR: periventricular; ROS: reactive oxygen species; TGFβ: transforming growth factor β

Since many individuals cannot exercise or achieve a certain level of intensity and duration of exercise, it is very important to study the molecular mechanism of exercise-promoted regeneration. Multiple mechanisms, including signaling pathways [7, 8], oxidative stress [3], metabolic reprogramming [9], and involvement of non-coding RNAs [10, 11], mediate the molecular response of exercise on tissue regeneration. Identifying novel regulatory molecular targets in exercise-driven regeneration has great significance, especially in translational research such as regenerative medicine. In this review, we discuss in detail the major effects of exercise on tissue regeneration in the muscle, the brain, and the blood vessel. Precursor cells or stem cells in these organ systems are activated by exercise to proliferate [7, 9], differentiate [12], or induce inflammatory responses that promote tissue regeneration [13]. We also describe the vital molecular mechanisms involved in exercise-mediated effects in different systems. This review aims to summarize the known mechanisms responsible for exercise-promoted tissue regeneration, which may lead to novel avenues for therapeutic interventions.

Given that exercise-mediated regeneration is an exhaustive topic, most of the data cited in this review were identified by searching PubMed and other references using the following keywords, alone or in combination: exercise, tissue regeneration, muscle stem cells, neural stem cells, cardiomyocytes, and regenerative therapy. While only articles published in English were included, abstracts and reports from meetings were excluded.

## Muscle Regeneration

Adult skeletal muscle consists of skeletal muscle fibers (muscle cells) and a small number of quiescent stem cells called muscle satellite cells or muscle stem cells (MSCs). MSCs were discovered in 1961 by Alexander Mauro and Bernard Katz [14]. These cells are located between the muscle membrane and the basal layer. They are small, in a quiescent state, and highly express Pax7. Lineage tracing experiments show that Pax7+ MSCs are activated to repair the injury and replenish the stem cell pool through proliferation, differentiation, and self-renewal [15]. The skeletal muscle is composed of multi-nucleated myocytes. Since the nuclei of skeletal myocytes are post-mitotic, the replenishment of myonuclei depends on the fusion of MSCs into a syncytium. Researchers have hypothesized that a myonucleus can only dominate a certain area of cytoplasm, the so-called myonuclear domain. Previous studies have shown that MSCs could contribute to the increase of myonuclei in response to physical activity, and the myonuclear number correlates with the size of myofibers, supporting the myonuclear domain theory [16]. However, recent reports in humans and rodents showed that type 2 muscle fibers could achieve hypertrophic growth without myonuclear accretion [17, 18], thus challenging this theory. Therefore, the role of MSCs in the myonuclear domain is highly context dependent and requires further investigation.

MSCs are essential for muscle growth after birth, but they enter a quiescent state in adults and are reactivated only in the event of muscle damage or exercise stimulation [19]. Upon muscle injury, the damaged extracellular matrix (ECM) releases growth factors such as FGF2 to stimulate the activation of MSCs by inducing a rapid intercellular increase of calcium concentration [20]. In fact, the FGF2 released by ECM increases cytosolic calcium levels through the transient receptor potential canonical (TRPC) channel, and the elevated calcium concentration triggers the nuclear translocation of the transcription factor nuclear factor of activated T-cell (NFATc), which in turn facilitates MSC activation. Interestingly, muscle regeneration also depends on MSCs migration. The injury at one end of the myofiber could activate all MSCs on the same muscle fiber and recruit these MSCs to the damaged sites [21]. The activated MSCs can migrate between muscle fibers during tissue regeneration, and this migratory ability of MSCs is regulated by Eph receptors and ephrin ligands [22]. Thus, various intrinsic and extrinsic factors regulate the MSC activation during the regeneration process. However, in certain conditions, such as aging, the regulatory factors of MSCs are disrupted, limiting their activation and regeneration. In the aging process, the regenerative potential of MSCs declines in response to the change in their niche. For example, the ligand SPARC-related modular calcium binding 2 (Smoc2) is affected by aging, and its aberrant expression leads to the impaired function of MSCs [23]. In addition, the aged ECM could induce the fibrogenic conversion of MSCs, which decreases the myogenicity of MSCs [24]. While cell transplantation experiments in mice have demonstrated that MSCs exhibit relatively robust stemness [25], they are less capable of self-renewal in the human body [26]. In 1990, an attempt was made to treat Duchenne muscular dystrophy (DMD) by transplanting muscle satellite cells [27]. Although the donor muscle satellite cell nuclei were detected in patients, the transplantation did not have a therapeutic effect, most likely due to the low survival and migration capacity of the grafted cells [28]. Therefore, the study of promoting muscle regeneration in situ has a certain value in regenerative medicine.

**Fig. 2:**
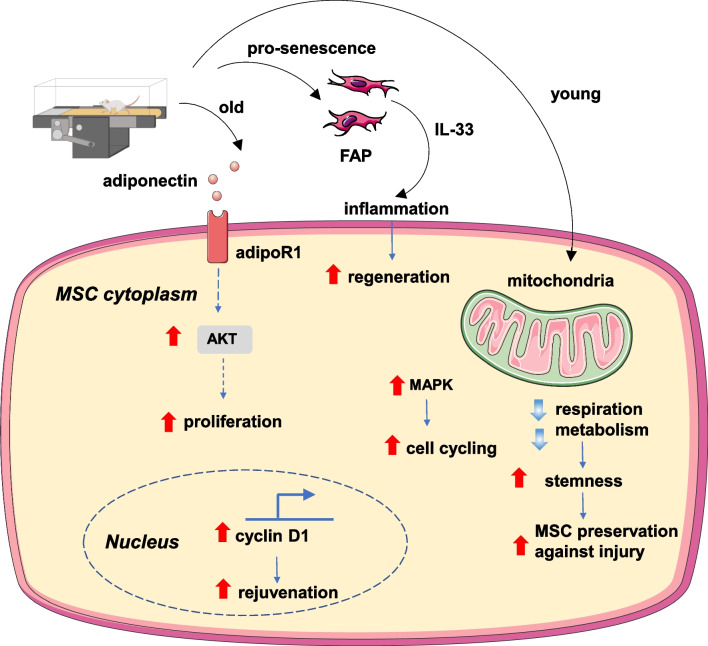
Main molecular mechanisms involved in exercise-induced muscular regeneration. adipoR1: adiponectin receptor 1; AKT: protein kinase B; FAP: fibro-adipogenic progenitors; MAPK: mitogen-activated protein kinase; MSC: muscle satellite cells; IL: interleukin

The protective effect of exercise on muscle atrophy is well recognized [29]. However, the effect of exercise on the proliferation and activation of MSCs is not fully elucidated. Research has shown that exercise-induced stem cell proliferation can repair muscle injury [7, 9] and promote muscle stem cell activation in aged mice [4, 8]. Moreover, exercise-induced muscle stem cell regeneration could repair chronic inflammatory myopathy [13]. Resistance training could produce mechanical overload on muscle and increase muscle mass, which is associated with elevated protein synthetic rate [30]. A recent study in elderly people showed that resistance training could increase knee extension strength and the number of MSCs in muscle fibers [31], suggesting a role for MSCs in the growth of myofibers. Mechanistically, signaling pathways, such as AKT and MAPK, as well as metabolic reprogramming, are involved in MSC proliferation and regeneration (Fig. 2). Interestingly, molecular regulators such as the AKT signaling pathway seem to play different roles in old and young mice. For example, long-term (4 months) exercise improved MSCs’ regenerative capacity by activating the AKT pathway and promoting the proliferation of MSCs in aged mice [8]. Another example of exercise-induced MSC rejuvenation is the restoration of cyclin D1 expression in aged mice, which promotes activation of MSCs [4]. In young mice, exercise promotes MSC cell cycling through the MAPK signaling pathway [7]. Further, besides promoting cell cycling, exercise also protects proliferative MSCs against exhaustion by inhibiting AKT-mTOR activity and mitochondrial metabolism which helps maintain the limited activation status of MSCs [7]. In young mice, exercise can promote satellite cell self-renewal by metabolic reprogramming pathways such as reduced mitochondrial respiration and increased stemness [9]. In addition to directly acting on MSCs, exercise can promote muscle regeneration through cellular cross-talk. For example, exercise can promote senescence of fibro-adipogenic progenitors (FAP) through AMPK signal activation [13], which activates muscle regeneration via regenerative inflammation. Interestingly, this study shows that exercise and pharmacological AMPK activation act synergistically during chronic inflammatory myopathy treatment. Summing up, exercise promotes muscle regeneration through various molecular mechanisms. Moreover, in muscle regeneration, the duration of exercise and factors such as age have a significant impact on the effect of exercise and interpretation of the underlying mechanism.

## Neural Regeneration

It is now recognized that two specific regions in the adult mammalian brain can continuously produce new neurons: the subventricular zone (SVZ) of the lateral ventricle and the dentate gyrus (DG) of the hippocampus. The latter region produces new neurons involved in regulating learning, memory, and emotion [32]. It was found that the neural progenitor cells (NPCs) in the SVZ do not have a neurogenic response to exercise, while NPCs in the DG of the hippocampus undergo significant proliferation after voluntary wheel running [33]. The DG of the hippocampus is a specific region that maintains adult neurogenesis. Neural progenitor cells in the subgranular zone of DG transform sporadically from a quiescent state to a proliferative state and participate in regulatory responses to physiological stimuli [34].

**Fig. 3:**
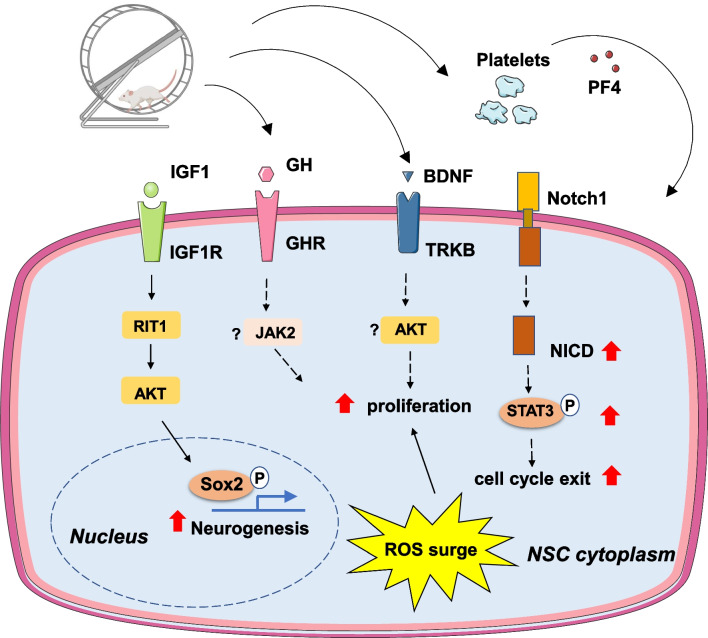
Main molecular mechanisms involved in exercise-induced neural regeneration. AKT: protein kinase B; BDNF: brain-derived neurotrophic factor; JAK2: Janus kinase 2; GH: growth hormone; GHR: growth hormone receptor; IGF1: insulin-like growth factor 1; IGF1R: insulin-like growth factor 1 receptor; NICD: notch intracellular domain; Notch1: notch receptor 1; TRKB: neurotrophic tyrosine kinase, receptor, type 2; PF4: platelet factor 4; RIT1: Ras-like without CAAX 1; ROS: reactive oxygen species; SOX2: SRY-box transcription factor 2; STAT3: signal transducer and activator of transcription 3

Research has shown that exercise could increase adult hippocampal NPC proliferation and neuronal differentiation [12, 35]. Moreover, exercise-induced NPC proliferation could repair the cognitive disorder of Alzheimer’s disease [36]. In addition to the hippocampus, exercise was also observed to promote the proliferation of periventricular (PVR) neural stem cells in aged mice [37], restoring NSC numbers to youthful levels. Exercise also increases oligodendrocyte precursor cells in the subventricular zone and ameliorates cognitive decline in hypo-perfused subcortical ischemic vascular dementia (SIVD) mice [38]. The role of exercise in synaptogenesis is also important for neuro-regeneration. Recent evidence has suggested that motor training requiring balance and coordination skills could facilitate synaptogenesis in the thalamus [39]. Treadmill exercise could increase spatial working memory in mice via strengthening synaptic plasticity in the hippocampus and prefrontal cortex [40]. In animal models of Parkinson's disease, exercise could affect synaptic connections and improve motor performance [41]. Mechanistically, growth factors such as vascular endothelial growth factor (VEGF) [42], insulin-like growth factor 1 (IGF1) [43, 44], growth hormone (GH) [37], and the neurotransmitter serotonin [34] are required for the increase in neurogenesis observed after exercise. Ras-related GTPase (Ras-like without CAAX 1, RIT1) acts as a downstream sensor of IGF1 in exercise-enhanced hippocampal neurogenesis via the IGF1–RIT1–Akt–Sox2 signaling pathway [44] (Fig. 3). A recent study found that exercise could improve brain-derived neurotrophic factor (BDNF) expression. This neurotrophic factor enhances neurogenesis and reduces inflammation in the brain affected by Alzheimer’s disease [36]. A previous study has suggested that BDNF has the potential to cross the blood–brain barrier [45]. Therefore, BDNF produced by other organs, such as skeletal muscle [46], may also contribute to exercise-induced neurogenesis. Interestingly, myokine cathepsin B could pass through the blood–brain barrier and promote BDNF generation and neurogenesis [47]. The metabolite lactate released by the muscles during exercise could also cross the blood–brain barrier and induce BDNF expression in the hippocampus [48]. Metabolic factors, such as PGC-1α and FNDC5, were shown to respond to exercise and promote BDNF expression in the brain [49]. Moreover, cellular redox states can regulate NPC activation. For example, in the hippocampus of adult mice, quiescent NPCs maintain the highest reactive oxygen species (ROS) levels (hiROS). Exercise recruits hiROS NPCs toward proliferation via a transient ROS surge [3]. The Notch pathway has been well recognized to play an essential role in adult NPC proliferation and maintenance [50]. Exercise-induced cell survival and cell cycle exit of type-3 progenitor cells could also be mediated by Notch1 activity [35]. The regulator of G protein signaling 6 (RGS6) was proven to be a key regulator that mediates the voluntary running-induced enhancing effect on adult neurogenesis [51]. RGS6 overexpression promotes the maturation of adult newborn neurons while knocking down RGS6 abolishes running-enhanced hippocampal neurogenesis. Interestingly, exercise could also regulate NSC proliferation via cellular cross-talk. For example, exercise activated platelets and promoted neurogenesis, mediated by platelet factor 4 (PF4) [12].

Summing up, although most studies on exercise response have focused on NPCs in the DG region, the observation of NPC proliferation in the PVR region in aged mice suggests that exercise response in NPCs of aged mice may be different compared to that of young mice, and this hypothesis requires further experimental validation. Even though the phenotype of exercise-enhanced NPC proliferation is apparent, and many beneficial regulatory molecules have been identified, the targets remain unclear, and the mechanism needs further clarification.

## Cardiac Regeneration

Cardiovascular disease is the leading cause of death worldwide [52]. The contraction of cardiac muscle cells brings about the expulsion of blood from the heart. Ischemic heart disease patients encounter considerable mortality of the cardiac myocytes, which have exited the cell cycle and cannot regenerate spontaneously. This results in a steady decline in cardiac function, ultimately leading to heart failure. So far, no cardiac stem cells have been identified in adults [53]. This has primarily hindered cell replacement therapy for the heart. Cardiac precursor cells induced by iPSC or embryonic stem cells can further differentiate into cardiomyocytes *in vitro* [54]. Transplantation of these induced cardiomyocytes is effective in non-human primates [55]. However, this technique has certain problems, such as low maturity and host immune rejection of differentiated cardiomyocytes. Direct promotion of endogenous cardiomyocyte regeneration can avoid those problems. Previous studies suggest that exercise can promote endogenous myocardial regeneration via promoting proliferation of existing cardiomyocytes [56]. In mice, voluntary wheel running and swimming promote the proliferation of existing cardiomyocytes [57]. Moreover, exercise can promote cardiomyocyte proliferation and heart repair after cardiac injuries, such as myocardial infarction (MI) [56, 58, 59], ischemia–reperfusion injury (IRI) [60, 61], pressure overload [62], and doxorubicin-induced cardiomyopathy [58]. Therefore, exercise has been recognized as an effective way to protect cardiovascular health and ameliorate cardiovascular disease (CVD) [63, 64].

**Fig. 4:**
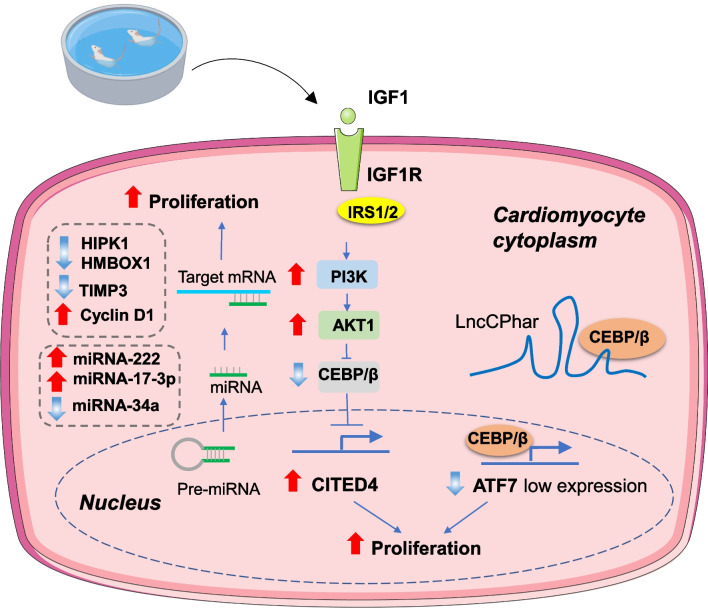
Main molecular mechanisms involved in exercise-induced cardiac regeneration. AKT1: protein kinase B; ATF7: activating transcription factor 7; CEBP/β: CCAAT enhancer-binding protein beta; CITED4: Cbp/p300-interacting transactivator with Glu/Asp-rich carboxyl-terminal domain 4; IGF1: insulin-like growth factor 1; IGF1R: insulin-like growth factor 1 receptor; HIPK1: homeodomain-interacting protein kinase 1; HMBOX1: homeobox containing 1; IRS1/2: insulin receptor substrate 1; PI3K: phosphatidylinositol 3-kinase; TIMP3: TIMP metallopeptidase inhibitor 3

Exercise-promoted cardiomyocyte proliferation could be mediated by the IGF–PI3K–Akt axis through downregulating C/EBPβ and upregulating CITED4 [62, 65, 66] (Fig. 4). Recent studies have shown that non-coding RNAs, acting as downstream regulatory molecules of physical activity, play an important role in exercise-mediated cardiac regeneration [67, 68]. MicroRNA (miRNA) is an endogenous single-stranded RNA molecule consisting of approximately 22 non-coding nucleotides. miRNA can bind to the untranslated region (3'-UTR) of the target gene mRNA, which leads to the inhibition of the target gene expression. Some miRNAs, such as miR-222 and miR-17-3p, participate in exercise-induced proliferation and regeneration of cardiac myocytes [57, 60, 69]. Moreover, exercise induced the expression of adenosine deaminase acting on RNA 2 (ADAR2) and enhanced the ADAR2–miR-34a–cyclin D1 axis-mediated regeneration of cardiac myocytes [58] (Fig. 4). Long non-coding RNA (lncRNA) is a class of RNA molecules with over 200 nt transcripts; usually, they do not encode proteins. LncRNA can bind to DNA/RNA or proteins to regulate biological functions. Previous studies have shown that C/EBPβ is a vital molecule mediating exercise-promoting regeneration [62]. A recent study has suggested that the long non-coding RNA LncCPhar responds to exercise and regulates cardiomyocyte proliferation by sequestering C/EBPβ [70] (Fig. 4). Another study showed that lncExACT exhibited decreased expression in hearts of mice undergoing exercise. Moreover, lncExACT1 inhibition could induce physiological hypertrophy mimicking exercise-enhanced cardio-myogenesis [71]. Taken together, non-coding RNAs such as miRNAs and lncRNAs play an important role in exercise-induced cardiomyocyte proliferation. Whether other non-coding RNAs, such as circular RNAs (circRNAs), play a role in exercise-induced cardiomyocyte proliferation needs to be further investigated.

## Angiogenesis and Lymphangiogenesis

Facilitating angiogenesis is essential for the tissue regeneration processes. The benefits of exercise include promoting angiogenesis and lymphangiogenesis [72]. Exercise activates the vascular endothelial growth factor pathway and improves cardiac angiogenesis in failing rat hearts [73]. Growing evidence suggests that lymphangiogenesis contributes to tissue repair and regeneration [74]. A recent study shows that swimming exercise-induced cardiac lymphangiogenesis contributes to physiological hypertrophy and proliferation of cardiomyocytes in mice [75]. Mechanically, exercise upregulates vascular endothelial growth factor receptor 3 (VEGFR3) level, and VEGFR3 activation is required for exercise-induced cardiac lymphangiogenesis. Endothelial progenitor cells (EPCs) are characterized as progenitor cells with angiogenic capacity in circulation. Interestingly, the EPC number in circulation was shown to be increased by exercise stimulation [76]. In humans, hypoxic exercise with low intensity has been shown to increase the number of vasculogenic cells and subsequently enhance skeletal muscle angiogenesis. Swimming training was shown to improve angiogenesis, increase the EPC number, and enhance EPC homing to the hindlimb ischemic sites in old mice [77]. Bone marrow transplantation can stimulate vascular regeneration and subsequently prevent muscle wasting in aged mice [78]. Interestingly, the neural protective effect of bone marrow mesenchymal stem cell transplantation could be enhanced by exercise training via the PI3K signaling pathway against spinal cord injury [79]. Moreover, moderate exercise has been shown to protect the brain against middle cerebral artery occlusion stroke via enhancing the function of EPC-derived exosomes [80]. EPC number and arterial elasticity decline with aging, while exercise increases circulating EPCs and arterial elasticity in aged men [81]. Summing up, exercise-induced EPC appearance in circulation and EPC homing could be beneficial for both angiogenesis and vascularization required for tissue regeneration.

### Regenerative Therapeutic Approaches

The previous sections of this review have interpreted various effects of exercise on different organ systems and their detailed molecular mechanisms. Although exercise has been proven to be an effective tissue regeneration stimulus, several individuals are not suited to perform certain types of exercise, which limits its therapeutic potential. Indeed, pharmacological approaches that target key pathways involved in exercise-induced regeneration may serve as an alternative strategy and have already shown some degree of protection.

#### Strategies Targeting IGF1

The IGF1 pathway is elevated in multiple organs in response to exercise. In the skeletal muscle, systemic IGF1 levels are rapidly increased in humans in response to resistance exercise [82, 83], which plays an important role in improving muscle strength [84] and promoting muscle satellite cell proliferation [85, 86]. Numerous studies have explored whether intrapericardial delivery of IGF1 or targeting IGF1 to injured tissue can mediate tissue regeneration. Sustained delivery of a combination of VEGF and IGF1 via injectable gel led to parallel angiogenesis and myogenesis in the context of ischemic muscle injury [87], suggesting the clinical potential of IGF1 treatment for muscle regeneration. In addition, increasing IGF1 via adeno-associated viral (AAV)-mediated gene therapy in skeletal muscle could increase muscle mass and strength as well as angiogenesis mimicking endurance exercise [88]. This gene-based approach can eliminate the risk of potential tumorigenicity induced by high levels of circulating IGF1, which may be applicable to treat degenerative muscle diseases.

In the adult brain, exercise stimulates the uptake of IGF1 to the hippocampus [43]. For example, swimming exercise could activate IGF1 signaling in the hippocampus and reduce aging-induced brain apoptosis [89]. In addition, resistance exercise training could increase IGF1 signaling pathway in the DG and ameliorate mild cognitive impairment [90]. Therefore, pharmaceutically enhancing IGF1 signaling in the brain may be protective against degenerative neural diseases. Indeed, adenoviral vector-mediated IGF1 gene therapy markedly restores neurogenesis of adult rats with spinal cord injury [91]. Moreover, AAV-mediated IGF1 and osteopontin overexpression in cortical neurons lead to robust corticospinal tract regrowth in spinal cord injured mice [92]. These findings suggest that IGF1 gene therapy is a potentially translatable strategy for restoring neuronal function in adults. Previous clinical trials using recombinant human IGF1 treatment in patients with amyotrophic lateral sclerosis have slowed the decline of motor functions [93, 94]. However, there are contradictory results showing no significant protective effect of the recombinant human IGF1 treatment [95, 96]. Therefore, a better delivery strategy with optimized dosage could greatly benefit the clinical application of IGF1 therapy against neural degenerative diseases.

IGF1 has been shown to regulate cardiomyocyte growth mediated by exercise and protect against CVDs [97, 98]. For example, swimming could effectively activate IGF signaling in the heart and induce protective physiological cardiac hypertrophy [99]. Therefore, enhancing cardiac IGF1 signaling might be beneficial in CVD patients. There have already been several attempts targeting IGF1 signaling in clinical trials [66, 100], but the therapeutic effects in patients with heart failure are controversial [101–105].Moreover, uncontrolled activation of the IGF1 pathway may cause tumorigenesis in other organs [106, 107]. Therefore, researchers seek novel strategies for activating the IGF1 pathway in the heart, such as delivering small molecules or naturally occurring compounds directly targeting damaged heart tissue. For example, BGP-15 is a small molecule that can increase the phosphorylation of IGF1R. Oral administration of BGP-15 for four weeks in mice with heart failure leads to improved heart function [108]. Because BGP-15 has a relatively short half-life, BGP-15 treatment may reduce the adverse effects of IGF1 ligand delivery. In another study, IGF1 was conjugated to Hoechst, which could bind to double-stranded DNA for targeting infarcted tissue [109]. Gene therapy, mediated by AAV serotype 9 expressing IGF1 Ea propeptide driven by the cardiac-specific promoter, was shown to be a clinically applicable treatment against ischemic injury [110]. Collectively, novel delivery methods combining small molecules and cardiac targeting vectors will greatly facilitate the development of IGF1-based regenerative therapy.

#### Strategies Targeting the PI3K–Akt Pathway

Exercise is a feasible strategy to prevent muscle atrophy via activating the PI3K signaling pathway and promoting myogenesis [111]. Multiple exercise conditions could help activate the PI3K signaling in muscle for regeneration. For example, aerobic and resistance exercises were shown to activate PI3K signaling in gastrocnemius muscle and alleviate skeletal muscle atrophy [111]. High-intensity intermittent exercise training was shown to enhance skeletal muscle angiogenesis via PI3K signaling [112]. These observations suggested a pivotal role of PI3K in myogenesis. Indeed, PI3K-p110α is necessary and sufficient for muscle stem cells to exit quiescence [113]. Transient downregulation of phosphatase and tensin homolog (PTEN) has been regarded as a promising strategy to accelerate tissue repair by activating P13K [114]. PTEN is a known negative regulator of the PI3K signaling pathway and regulates cell proliferation [115]. Pharmacological inhibition of PTEN leads to PI3K activation and improved tissue repair [116]. Bisperoxovanadium is a relatively specific PTEN inhibitor that shows beneficial effects on the muscle repair process [117–119], all of which indicates the feasibility of pharmacological PI3K activation mimicking the beneficial effects of exercise in muscle regeneration.

Exercise conditions were shown to activate PI3K signaling in the brain. For example, swimming training could effectively activate PI3K signaling in the hippocampus of aged rats [89]. In addition, the reduced PI3K signaling in aged rats could be restored by resistance exercise and treadmill running [120]. A recent study reveals that treadmill running-enhanced neuronal progenitor cell growth could be mediated by serum extracellular derivatives (EDs) targeting the PI3K–Akt pathway [121]. This study shows that running-induced serum EDs could increase cell viability and Akt phosphorylation. Therefore, exploring the specific molecule in the exercise-induced EDs responsible for this protective effect of exercise will facilitate the development of neural regenerative drugs targeting the PI3K–Akt pathway. Several studies have already focused on the use of the PI3K pathway to treat brain injury [122]. For example, formononetin prevents ischemia or reperfusion injury of the brain by activating the PI3K pathway [123]. In the peripheral nervous system, exogenous FGF10 treatment could activate PI3K/Akt signaling and promote axonal regeneration after nerve damage [124]. Collectively, these findings provide promising therapeutic drugs for neural regeneration targeting PI3K/Akt signaling.

In the adult heart, exercise conditions stimulate the PI3K signaling for cardio-protection. For example, PI3K was shown to be necessary for swimming-induced cardiac beneficial effects [125]. In addition, treadmill exercise could activate PI3K signaling and induce cardio-protection in adult rats with myocardial infarction [126]. High-intensity interval swimming has also been shown to induce protective physiological hypertrophy in rats, and this exercise condition may be more beneficial due to shorter training time [127]. These observations suggest the possibility of PI3K as a target in CVD treatment. AAV vector carrying constitutively activated phosphatidylinositol 3-kinase (caPI3K-p110α) was used to achieve selective expression in the cardiac myocytes of adult mice [125]. The recombinant AAV serotype 6 (rAAV6) construct containing a cytomegalovirus (CMV) promoter encoding caPI3K (rAAV6-CMV-caPI3K) induced physiological hypertrophy in mice. Furthermore, rAAV6–CMV–caPI3K delivery improves systolic heart function in mice with cardiac pressure overload induced by TAC [125]. Additionally, PI3K gene therapy also improves the diastolic function of mice with diabetic cardiomyopathy [128, 129], suggesting the clinical potential of PI3K gene therapy in treating CVDs.

### Exercise-Regulated miRNAs with Proliferative Effects

Exercise training in mice has been shown to stimulate cell proliferation in multiple organs, mediated by microRNAs. Physical exercise could increase miR-23a and miR-27a levels in mice with muscle atrophy caused by chronic kidney disease (CKD) [130], and overexpression of miR-23a/miR-27a via adeno-associated virus in vivo attenuated muscle loss in CKD mice. Exercise could alleviate muscle atrophy via downregulation of miR-29b [29]. Indeed, suppression of miR-29b could prevent angiotensin II (AngII) induced muscle atrophy in mice [131], and its potential therapeutic application has been explored [132, 133]. Collectively, these findings prove the therapeutic potential based on exercise-regulated microRNAs in muscle regeneration.

In the mouse DG, exercise could increase the proliferation of neural precursor cells (NPCs) via the downregulation of miR-135a [134]. MiR-135a inhibition stimulates NPC proliferation and neurogenesis via inositol 1,4,5-trisphosphate (IP3) signaling [134]. Therefore, miR-135a inhibition may represent a novel therapeutic intervention to promote NPC proliferation in DG. Interestingly, miR-135a was shown to promote axon regeneration in the spinal cord [135] and enhance ganglion cell axon regeneration in the retina [136], suggesting distinct roles and targets of miR-135a in different neuronal cell types.

Several miRNAs have been reported to promote cardiomyocyte proliferation and are regulated in the heart by exercise [10, 11]. miR-17-3p, which contributes to exercise-induced cardiomyocyte proliferation, has also been shown to have protective effects. Mice treated with a miR-17-3p agomiR to increase miR-17-3p expression showed preserved cardiac function and increased markers of cardiomyocyte proliferation [60]. Transgenic overexpression of miR-222 protected the heart against ischemic injury [57]. Moreover, treatment of exosomes from adipose-derived stem cells (ADSC-Exo) prevented cardiac ischemia/reperfusion (I/R) injury via increasing miR-221/222 expression [137]. These observations indicate that pharmacologically increasing exercise-induced cardiac protective miRNAs such as miR-222 may serve as a protective approach against CVD. Summing up, the studies above suggest that targeting signaling pathways and miRNAs mimicking the protective effects of exercise may prove to be a promising approach for developing novel regenerative therapy.

## Conclusions

Although many studies suggest that exercise plays a positive role in promoting adult tissue regeneration, some questions remain unanswered and should be considered for the future, such as: (1) Factors such as age may cause variations in the effect of exercise. Therefore, the phenotype should be more carefully evaluated in studies involving age and other influencing factors. (2) Exercise may not be sufficient on its own. Under some circumstances, exercise acts as a cofactor in coordination with other regenerative factors. Exogenous supplementation of pro-regeneration molecules during exercise may be a tentative strategy to promote regeneration. (3). Regulatory molecules of exercise may have opposite effects on different types of cells. Therefore, when applying exogenous transfection, the precision of targeting is very important. (4). Although the phenomenon of exercise-promoted regeneration is well established in some organs, the molecular mechanisms remain to be further explored. In summary, the discovery of novel molecules mediating the pro-regenerative effects of exercise will contribute toward developing new therapeutic strategies in regenerative medicine and the effective treatment of degenerative diseases.

## Data Availability

Not applicable.

## Abbreviations

ADAR2: Adenosine deaminase acting on RNA 2
AKT: Protein kinase B
AMPK: AMP-activated protein kinase
BDNF: Brain-derived neurotrophic factor
circRNAs: Circular RNAs
CITED4: CBP/p300-interacting transactivators with E [glutamic acid]/D [aspartic acid]-rich carboxyl-terminal domain 4
DG: Dentate gyrus
DMD: Duchenne muscular dystrophy
FAP: Fibro-adipogenic progenitors
lncRNA: Long non-coding RNA
mTOR: Mammalian target of rapamycin
MAPK: Mitogen-activated protein kinase
MSCs: Muscle stem cells
NPC: Neural precursor cell
Nox: NADPH-oxidizing enzyme
ROS: Reactive oxygen species
SVZ: Subventricular zone
